# Description of the modified vestibular incision subperiosteal tunnel access (m-VISTA) technique in the treatment of multiple Miller class III gingival recessions: a case series

**DOI:** 10.1186/s12903-021-01511-5

**Published:** 2021-03-20

**Authors:** Aitziber Fernández-Jiménez, Ruth Estefanía-Fresco, Ana-María García-De-La-Fuente, Xabier Marichalar-Mendia, Luis-Antonio Aguirre-Zorzano

**Affiliations:** 1 Department of Stomatology II, University of the Basque Country (UPV/EHU), UPV/EHU. Barrio Sarriena S/N, 48940 Leioa, Biscay Spain; 2 Department of Nursing I, University of the Basque Country (UPV/EHU), Leioa, Biscay Spain

**Keywords:** Gingival recession, Mucogingival surgery, Connective tissue graft, Periodontal plastic surgery

## Abstract

**Background:**

Gingival recession is a common finding in the adult population. It is considered a challenge for clinicians to obtain a complete root coverage of Miller class III recession. The aim of this case series was to assess the outcomes achieved with the use of modified VISTA technique (m-VISTA) in patients having multiple Miller class III recessions after 6 months.

**Methods:**

Ten patients (six women and four men; mean age: 53 years), who showed multiple Miller class III recessions (depth ≥ 2 mm) and who met the established inclusion and exclusion criteria, were treated by postgraduate students with the use of m-VISTA technique.

**Results:**

A total of 38 recessions were performed. The recessions were mainly located in the mandible (80%), which included six molars. The mean baseline recession was 3.12 mm. Post the intervention, a mean root coverage of 58.72% was achieved, with complete root coverage observed in 29% of the recessions.

**Conclusions:**

m-VISTA may offer several advantages in the treatment of Miller class III gingival recession. Nevertheless, more clinical trials with a longer follow-up period are needed to arrive at a concrete conclusion about its advantages.

*Trial registration*: NCT03258996.

*Data registration*: 08/18/2017.

**Supplementary Information:**

The online version contains supplementary material available at 10.1186/s12903-021-01511-5.

## Background

Periodontitis is considered to be the sixth most prevalent disease worldwide [1]. If not treated, it may lead to the destruction of the periodontal soft and hard tissues, which in turn may lead to the root surface being exposed to the oral environment, and multiple gingival recessions showing up in patients with periodontitis [2]. Gingival recession is a common finding in adult patients, and its prevalence increases with age [3]. Gingival recession is classified as Miller class III recession [4] or RT2 [5] depending on the interproximal attachment loss and/or the malposition of the tooth [4], which could hinder the surgical attempt to achieve complete root coverage (CRC) [4, 6]. These lesions could not only have an undesired esthetic outcome, but could also cause hypersensitivity, root caries, or non-carious cervical lesions, which may impact a patient’s quality of life to a variable degree [7].

Over the last few decades, multiple surgical approaches have been described for the treatment of this type of recession [8, 9]. These techniques are similar to those used for Miller class I/II [4] recessions. Their aim is to obtain a complete root coverage of the gingival recession. In the recent past, CRC of Miller class III recession has come to be reported as a real treatment outcome possibility [10], for only a partial root coverage (RC) has come to be expected [11]. Nevertheless, from a clinical point of view, achieving CRC in Miller class III recession might not be a realistic objective. To attain a partial RC with an increased amount of attached gingiva can be considered as a successful treatment outcome [9, 11]. The literature fails to define the principal parameter to assess the success of the treatment, or fails to answer if the success rate should be determined by a single or a combination of various parameters.

Until now, the evidence regarding root coverage in Miller class III recessions [4] has been limited to multiple case reports and case series [8], four retrospective studies [12–15], and ten randomized clinical trials (RCTs) [16–25]. Only three of the RTCs’ [17, 24, 25] multiple Miller class III gingival recessions [4] (a total of 501 recessions) were treated, with the follow-up period ranging from 6 to 36 months [16–25]. Having taken into consideration all the randomized clinical trials, these trials showed heterogeneous results: the mean root coverage (MRC) ranged from 56.78% [19] to 95.10% [22] and the CRC ranged from 13.30% [23] to 74.20% [22]. The trials mainly used coronally advanced flap (CAF) techniques, which were combined with several variations of grafts, namely connective tissue graft (CTG) [19, 20, 22, 24], enamel matrix derived proteins (EMD) [16, 19, 24], recombinant human platelet-derived growth factor-BB (rhPDGF-BB) [25], and acellular dermal matrix (ADM) [18].

While CAF [8] has been the most frequently used technique in the treatment of multiple gingival recession [26], new minimally invasive techniques, such as *vestibular incision subperiosteal tunnel access* (VISTA) [27], have been suggested. The VISTA technique [27] consists of performing a vertical vestibular incision in the mucosa, usually at the level of the maxillary frenulum. Subsequently, the elevation of the subperiosteal tunnel is continued through the vertical incision. It should extend over to the gingival margin of at least one tooth adjacent to the teeth requiring RC [27]. While this technique was initially prescribed for the treatment of Miller class I and II recession [4] in the maxilla, it can also be applied for other locations. This approach, to preserve the vascularization of the area to be treated, avoids the incision or traumatization of the marginal gingival tissues. Regardless of the surgical approach, CTG is considered as the gold standard graft in RC techniques [8].

The aim of this case series is to present the modified VISTA (m-VISTA) technique in a step-by-step manner and describe the results that are obtained in 6 months after the treatment of multiple Miller class III recessions [4] (including more than two recessions). The main modifications consist of extending the vertical incision slightly beyond the mucogingival line, performing intrasulcular incision, and releasing the tunnel-papillae complex completely to facilitate the coronal traction of the whole tunnel-graft-papillae complex.

## Methods

A total of 10 patients (6 women, mean age being 53 years [41–61]) were consecutively enrolled between January 2018 and February 2019 after obtaining their informed consent. All patients included in this clinical study were explored clinically and radiographically to establish a precise periodontal diagnosis. Patients who showed multiple Miller class III recessions [4] with indications of requiring treatment and who met the inclusion criteria were invited to participate. All patients were treated by students who were pursuing master’s in Periodontics and Osteointegration at the University of the Basque Country (UPV/EHU). These patients were a part of an ongoing RCT, in which m-VISTA was compared to CAF for the treatment of multiple Miller class III recessions [4]. The data from this subset of patients were gathered and analyzed to introduce this modified technique. This study was performed in accordance with the Helsinki Declaration of 1975, as revised in Tokyo 2004, and received the approval of the Ethics Committee for Research of the University of the Basque Country (UPV/EHU) (CEISH/M10_2017_042). This study’s ClinicalTrials.gov identifier is NCT03258996.

Patients presenting multiple Miller class III recessions [4] (> 2 recessions) with a depth of ≥ 2 mm were considered eligible for this study if they met the following inclusion criteria: age ≥ 18 years, absence of active periodontal disease, and presence of plaque [28] and bleeding [29] indices ≤ 25%. Patients were excluded if they smoked > 10 cigarettes/day, had systemic conditions contraindicating surgical treatment, and were pregnant or nursing women.

All measurements were recorded by the same trained, blinded, and calibrated examiner (R.E.) using a calibrated periodontal probe (PCP-11, Hu-Friedy, Mfg. Co. LLC, Chicago, USA). Calibration analysis was performed by measuring the multiple recessions of four patients who did not take part in the study during the two different visits, which were at least 24 h apart. The intraclass correlation coefficient was > 0.75%. The following clinical parameters were assessed at baseline and 6 months after the surgery: probing depth (PD; distance in mm from the gingival margin to the bottom of the periodontal pocket), gingival recession (REC; distance in mm from the cementoenamel junction to the gingival margin), width of the gingival recession (GRW; mesiodistal distance of the recession; measured in mm at the most coronal point), width of the keratinized gingiva (KGW; distance in mm from the mucogingival junction to the gingival margin; measured in mid-buccal), distance from the contact point to the interdental papilla (CP-IP; distance in mm from the mesial and distal contact points of the tooth with recession to the most coronal part of the interdental papilla), patient’s full mouth plaque index (FMPI) [28], and full mouth bleeding index (FMBI) [29]. CRC was assessed at the 6-month follow-up, recording the number of treated recessions whose REC = 0 mm.

The patients’ perceptions of acute post-surgical pain were assessed using a pain diary designed by the UPV/EHU. An additional file presents this in more detail (see Additional file 1). The pain diary was given to the patients on the day of surgery. They were given precise instructions on how to fill it. The highest intensity of perceived pain was recorded by using a visual analogue scale (VAS) (0–100 mm). The recording was done 2 and 4 h after the surgery during the first 24 h, every 8 h the following 2 days, and daily, late in the evening, during the week, or until the pain disappeared. In addition, the Central Sensitization Inventory (CSI) [30] was used prior to the surgery to record the patients’ CSI severity levels, which ranged from subclinical, mild, or moderate to severe or extreme scores.

In addition, the patients’ perceptions of the esthetic outcome was assessed 6 months after the surgery, which ranged from non-esthetic (VAS = 0) to the most likely esthetic outcome (VAS = 100). Finally, the presence or absence of postsurgical complications (PSCs) was recorded.

## Surgical technique: modified VISTA (m-VISTA)

**Fig. 1:**
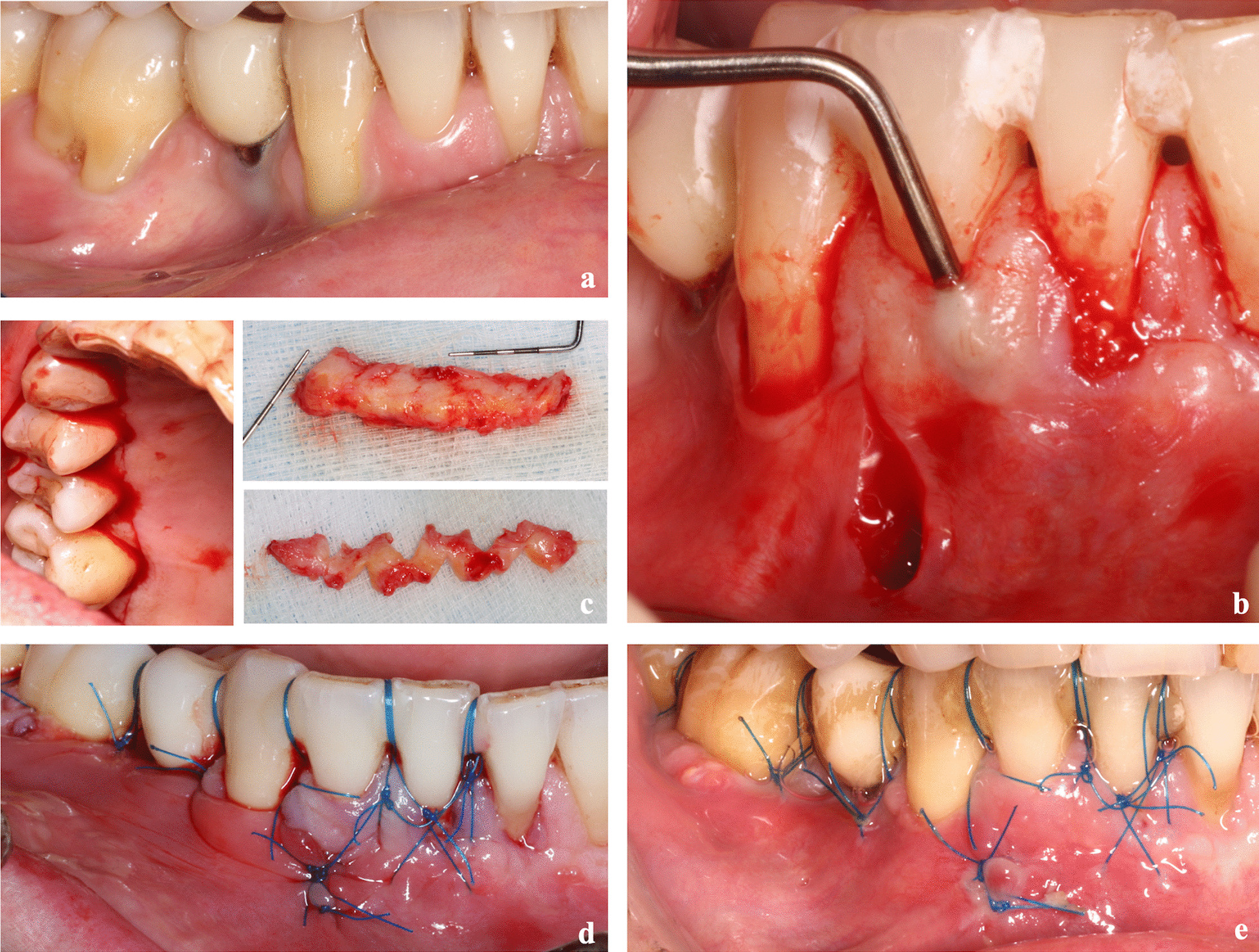
**a** Initial situation. **b** After the preparation of the denuded root surface and the placement of composite in the interproximal sites, a single vertical incision in the mucosa and intracrevicular incisions were performed, preparing a full-thickness tunnel and raising the papillae. **c** The CTG harvested from the palate using the UPV/EHU technique and its dimensions increased with the expanded mesh CTG procedure. **d** After the placement of CTG through the vertical incision, vertical double-crossed sutures were performed, coronally tractioning the tunnel-graft-papillae complex as well as the single interrupted sutures in the vertical incision. **e** 14 days of postoperative healing

Patients were instructed to rinse their mouths with a mouthwash (0.12% of chlorhexidine digluconate) for a minute prior to the surgery (Fig. 1a). After the administration of local anesthesia, exposed root surfaces were scaled and planned using Gracey curettes (Hu-Friedy, Mfg. Co. LLC, Chicago, USA) and a diamond bur (PerioSet®, Vanetti S.A., Gordevio, Switzerland). To facilitate the anchorage of sling sutures when suturing, flowable composite (Tetric EvoFlow®, Ivoclar Vivadent S.L.U., Madrid, Spain) was placed (with no etching) in the mesial and distal sites of the teeth to be treated.

The modified technique started with a single vertical incision [27] of sufficient length. The incision extended to the periosteum and went slightly beyond the mucogingival junction. Considering the extension of the teeth to be treated, a vertical incision was made in the most centered section. Subsequently, all intracrevicular incisions were performed with a microsurgical blade (SM69®, Swann-Morton Ltd, United Kingdom), except for the incisions of inferior incisors, where a smaller, disposable blade (KAI®, Kai Europe GmbH, Solingen, Germany) was used. These incisions were extended to at least one of the teeth that were beyond those to be treated and towards the base of the papillae.

A full-thickness tunnel was prepared with the aid of specific tunneling instruments (Stoma®, Ancladen S.L., Barcelona, Spain), extending it sufficiently beyond the mucogingival line into the alveolar mucosa. This is done first through a vertical incision and then through gingival margins, completely releasing the tunnel-papillae complex, thus facilitating its passive coronal replacement (Fig. 1b).

Next, a CTG with a thickness of approximately 2 mm and a sufficient length to cover the lesions was harvested on the same side of the palate using the UPV/EHU technique [14] (Fig. 1c). The “UPV/EHU technique” [31] begins with the elevation of the full thickness flap (FTF) in the palate and an intrasulcular incision performed with a number 12 blade, preserving the papillae in the interproximal spaces. Then, the FTF is dissected with a 15c blade, holding the flap with tissue forceps, leaving the epithelium and a thin layer of the connective tissue in the flap, so that the underlying connective tissue can be harvested. When required and owing to the extension of the treated areas, the CTG dimensions were increased using the expanded mesh CTG (e-MCTG) procedure [32] (Fig. 1c). While the donor site was sutured, the CTG was kept in a saline solution.

By using the positioning suture, the CTG was placed through the vertical incision. The needle was inserted in the most distal gingival sulcus, crossing the tunnel and coming out through a single vertical incision. Following the piercing of the graft, it returned through the tunnel following the same route. The same procedure was repeated in the mesial direction. The graft was easily and precisely placed in the recipient site with the assistance of a periostotome and by pulling the suture slightly.

The graft and tension-free tunnels were simultaneously sutured and coronally replaced. The suturing of both the CTG and the flap with vertical double-crossed sutures [33], anchored at each contact point, would ensure close adaptation of the tissues and compression of the wound. Finally, single interrupted sutures were used for the vertical incision (Fig. 1d).

The post-surgical protocol included the following steps: (a) taking amoxicillin 875 mg/clavulanic acid 125 mg (Augmentine®, GlaxoSmithKline S.A., Madrid, Spain) orally every 8 h for 7 days; (b) taking ibuprofen 400 mg (Ibuprofeno Kern Pharma®, Kern Pharma, S.L., Barcelona, Spain) orally every 8 h for 2 days; (c) carrying out mouthwashes with chlorhexidine digluconate 0.12%, twice a day for 6 weeks; (d) avoiding the brushing of the surgical site during the first 3 weeks post the intervention; (e) local application of cold for 2 days; and (f) having a soft diet, avoiding trauma in the treated area and avoiding physical exercise during the first week after the surgery. According to the protocol, ibuprofen had to be taken for 2 days. The patients were instructed to lengthen this period if necessary and record this additional medication intake in the pain diary.

Sutures were removed from the palate and the recipient site for weeks 1 and 2 after the surgery (Fig. 1e). The patients were instructed to restart oral hygiene in the third week post-surgery using the Stillman technique with an ultra-soft brush. Six weeks after the surgery, the patients resumed their dental and proximal hygiene habits. Finally, all the patients were enrolled into a supportive periodontal therapy program, with there being a reinforcement of oral hygiene after the intervention at 1, 3, and 6 months.

**Fig. 2:**
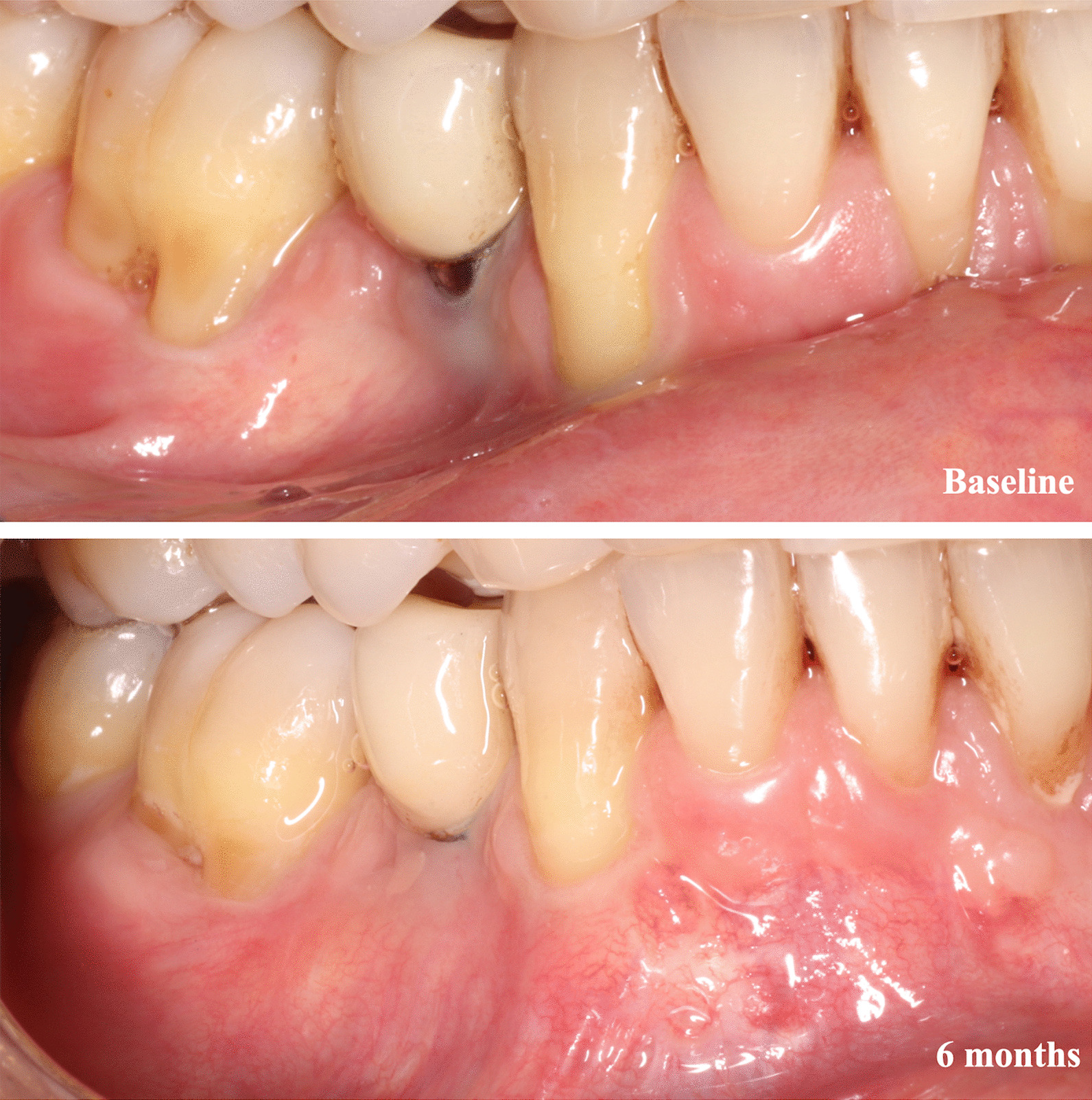
Initial situation and healing at 6 months after surgery

A blinded statistician (X.M.) performed the statistical analysis using IBM® SPSS® Statistics 20 software. A patient was considered as the unit of analysis. Clinical attachment level (CAL) was calculated (CAL = PD + REC) at the baseline at the 6 months mark (Fig. 2) with the following variables: mean root coverage (MRC = mean REC at baseline—mean REC at 6 months), the percentage of MRC (MRC% = mean preoperative REC-mean postoperative REC/mean preoperative REC × 100), the percentage of CRC (CRC% = number of locations with CRC × 100/number of recessions), and the variables of change of the parameters registered in both baseline and 6-month visits (PD, CAL, GRW, KGW, and CP-IP).

The normal distribution of the quantitative variables was confirmed with the Shapiro–Wilk test. Descriptive statistics were used to calculate the mean and standard deviation of the normally distributed variables or the median and interquartile range of variables that were normally not distributed. The frequencies of the categorical variables were also calculated. The statistical significance was set at *p* < 0.05.

## Results

Four men and six women participated in this case series, with the average age of patients being 53.68 years [41–61]. Four patients had at least one systemic disease. While one of the patients was a light smoker (< 10 cig/day), none of them were alcoholic or drug abusers. All patients showed good oral hygiene, with the mean plaque control (FMPI) being 13.50 ± 6.69% and the bleeding index (FMBI) being 8.94 ± 4.43% (Table 1).

**Table 1:** Characteristics of the study population

Patients	Age (years)	Systemic disease	Drugs	Smoker		
				Type	Cig/day	Years
1	56	Fibromyalgia Migraine	Citalopram Lorazepam	FS	0	3
2	41	Hypercholesterolemia Arthrosis	No	NS	0	0
3	50	No	No	FS	0	1
4	57	No	No	NS	0	0
5	60	Renal insufficiency Asthma Hypercholesterolemia	Terbutaline Rocatrol	FS	0	6
6	51	No	No	S	9	17
7	51	No	No	FS	0	27
8	51	No	No	NS	0	0
9	61	No	No	NS	0	0
10	54	Asthma Depression	Terbutaline Budesonide/formoterol Escitalopram	FS	0	6

Cig/day, cigarettes/day; FS, former smoker; NS, non-smoker; S, smoker

A total of 38 recessions were treated, with every participant presenting at least 3 recessions. The recessions were primarily located in the mandible (80%), which included six molars. These recessions showed a mean PD of 1.80 ± 0.52 mm, a mean REC of 3.12 ± 0.89 mm, a mean CAL of 4.93 ± 1.29 mm, a mean GRW of 4.37 ± 1.13 mm, a mean KGW of 2.63 ± 1.22 mm, and a mean CP-IP of 2.51 ± 1.46 mm (Table 2).

**Table 2:** Intraoral characteristics of the study population

Patients	FMPI (%)	FMBI (%)	n	Localization		Recession				
						PD (mm)	REC (mm)	GRW (mm)	KGW (mm)	CP-IP (mm)
				Arch	Tooth					
1	20.67	6.00	4	Md	3.1, 3.2, 3.4, 3.5	1.25	3.25	3.25	2.00	2.50
2	11.90	17.26	5	Md	4.6, 4.5, 4.4, 4.2, 4.1	1.20	2.40	3.80	2.80	3.10
3	15.53	11.11	3	Md	4.6, 4.3, 4.1	2.67	5.00	5.00	1.00	4.50
4	4.17	3.47	3	Md	3.4, 3.5, 3.6	2.00	4.33	6.33	2.67	3.00
5	13.89	9.72	4	Mx	2.1, 2.2, 2.3, 2.5	1.00	2.50	2.75	3.00	3.43
6	19.57	11.59	4	Md	3.2, 3.3, 3.4, 3.5	2.00	3.00	4.25	2.25	1.17
7	24.36	13.46	4	Mx	2.1, 2.2, 2.3, 2.4	2.00	2.50	3.25	4.75	4.33
8	6.79	4.32	3	Md	4.6, 4.5, 4.4	1.67	2.33	5.33	4.33	0.33
9	12.18	6.41	4	Md	4.6, 4.5, 4.4, 4.3	2.00	2.75	4.50	1.00	0.50
10	6.00	6.00	4	Md	4.6, 4.5, 4.4, 4.3	2.25	3.25	5.20	2.50	2.25
Mean [Range]						1.80 [1.00–2.70]	3.12 [2.33–5.00]	4.37 [2.75–6.33]	2.63 [1.00–4.75]	2.51 [0.33–4.50]

FMPI, Full Mouth Plaque Index; FMBI, Full Mouth Bleeding Index; Mx, Maxilla; Md, Mandible; PD, Probing Depth; REC, Recession; GRW, Gingival Recession Width; KGW, Keratinized Gingiva Width; CP-IP, Distance from the Contact Point to the Interdental Papilla

By using the m-VISTA technique, an MRC of 58.72 ± 25.95% was achieved. CRC was seen in 29% of the recessions and in 50% of the patients. Furthermore, a CAL gain of 1.76 ± 1.07 mm, a KGW gain of 1.11 ± 1.04 mm, a GRW reduction of 2.26 ± 1.25 mm, and a CP-IP reduction of 0.80 ± 1.06 mm were also recorded (Table 3).

**Table 3:** Recessions characteristics at 6 months after the intervention with the m-VISTA technique

Patients	PD (mm)	REC (mm)	CRC (n)	GRW (mm)	KGW (mm)	CP-IP (mm)
1	1.50	2.50	0	3.00	2.50	1.00
2	1.40	1.60	4.2 (1)	2.80	4.60	1.90
3	2.00	2.33	0	3.67	3.33	3.17
4	2.00	1.33	0	4.33	4.00	1.50
5	1.50	0.50	2.1, 2.2, 2.3 (3)	0.50	5.50	3.20
6	2.25	0.50	3.3, 3.4 (2)	1.50	2.00	1.83
7	1.75	1.25	0	1.25	6.75	1.50
8	2.00	1.67	0	2.00	4.00	0.67
9	2.25	0.00	4.3, 4.4, 4.5, 4.6 (4)	0.00	2.00	0.25
10	2.25	1.00	4.5 (1)	2.00	2.75	2.13
Mean [range]	1.89 [1.40–2.25]	1.27 [0.00–2.50]	11 [0–4]	2.11 [0.00–4.33]	3.74 [2.00–6.75]	1.72 [0.25–3.20]
Change Baseline-6 months (SD)	0.09 (0–34)	− 1.85 (0.92)		− 2.26 (1.25)	1.11 (1.04)	− 0.80 (1.06)

PD, Probing Depth; REC, Recession; CRC(n), Number of recessions with complete root coverage; GRW, Gingival Recession Width; KGW, Keratinized Gingiva Width; CP-IP, Distance from the Contact Point to the Interdental Papilla; SD, Standard deviation

The CTGs harvested from the palate showed a mean length of 29.92 ± 7.83 mm, a mean width of 7.31 ± 1.76 mm, and a mean thickness of 2.55 ± 0.89. E-MCTG [32] was performed in 7 of the 10 patients. No significant post-surgical complications were recorded, with only 4 patients showing mild complications: facial hematoma (n = 1) and herpes simplex virus (n = 3).

**Fig. 3:**
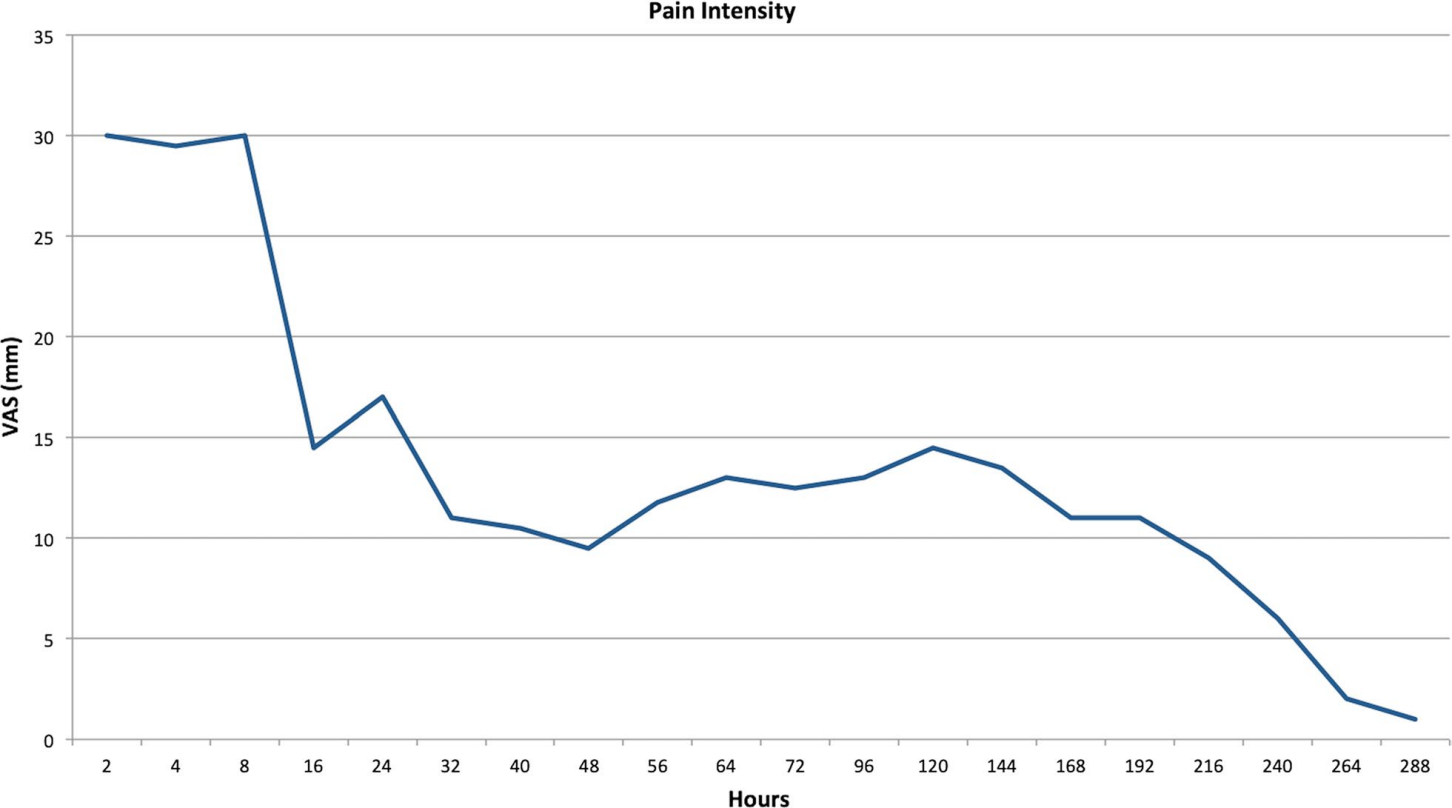
Diagram of the mean post-surgical pain experienced by patients at different time-points

With regard to the patients’ perception of pain, the mean VAS intensity of pain experienced was 13.51 ± 12.86 [2.00–38.50]. After the first day post-surgery, nearly half (four) of the patients had no pain. Only two patients were referred to have had pain 1 week after the surgery (Fig. 3). The average duration of the pain was 74.33 ± 140.74 min [1.60–450.00]. While half of the patients required supplemental analgesia, only two of them needed more than one additional intake of the previously established medication.

In addition, while assessing the patients’ perceptions of the esthetic result 6 months after the surgery, the mean VAS score was found to be 81.90 ± 17.30.

## Discussion

At present, the evidence concerning the treatment of Miller class III [4] gingival recession is scarce. The evidence is mainly based on case reports [8], four retrospective studies [12–15], and ten clinical trials [16–25]. This scarcity could probably be, not because of the fact that these recessions are not being treated, but because the predictability of outcomes in the treatment of this type of lesion is low. This would not always be as good as desired if the primary outcome variable was CRC.

In this case series, 10 patients with multiple Miller class III [4] gingival recession were treated by using a modification of the original VISTA technique [27]. Four modifications were included in the m-VISTA technique. First, the confection of interproximal composite stitches prior to the preparation of the surgical bed, thus reducing the surgical time. The intra-surgical time would influence the healing, the results of root coverage, and the postoperative state of the patient. Second, the execution of a vertical incision in the middle of the intervened area, which extends slightly beyond the mucogingival line, would facilitate the coronal replacement of the inserted gingiva, the most complicated area to be coronally replaced in a tunnel preparation. Third, performance of intrasulcular incisions, which extend to the papillae, might facilitate the coronal replacement of the whole tunnel-graft-papilla complex. Coronal traction of the papilla provided greater lateral vascularization of the graft at the marginal level, for we started from an initial situation of loss of interproximal insertion. The underlying connective graft could provide greater stability to the gingival margin, obtaining better RC results [34]. Fourth, performing multiple vertical double-crossed sutures [33] on the interdental composite stitches would ensure the complete traction of each gingival margin and minimize the possible risks that are associated with a single suture.

As a matter of fact, while the recessions were treated by postgraduate students, an MRC of 58.72% and a CRC of 29% were achieved in the locations. These outcomes are within the range described in the literature for the treatment of any type of multiple recessions (MRC% ranged between 57 and 97%; CRC% ranged between 24 and 89%) [35], and for both single and multiple Miller class III recessions (MRC% ranged from 61.3% to 86.4% and CRC% ranged from 0 to 50%) [36]. As previously mentioned, different approaches have been described to treat Miller class III recessions, including CAF, tunnel technique, and lateral flaps [8]. Among them, CAF with CTG alone or in combination with EMD has been the most tested and successful technique in Miller class III recession [8], achieving the best MRC percentages based on the site-recession that ranged from 62.83% [19] to 64.57% [24] with the CRC of 14.17% [24]. Aroca et al. [17] reported a greater MRC (82.09%) at the six months mark. However, a modified tunnel technique was utilized to explain the differences in the results obtained by Henriques et al. [19] and Mercado et al. [24]. To the best of our knowledge, the VISTA technique or its modifications have not been evaluated prospectively or in an RCT. It seems as though different surgical approaches could determine the RC.

With respect to MRC, our results were more modest than those reported by a recent study [14], in which Miller class III recessions [4] treated with the VISTA technique [27] were retrospectively analyzed (84.30%). To achieve success when treating this type of recession, one key factor is the surgeon’s skills and experience [18]. This could explain the differences observed when comparing the coverage results described in the literature to those reported in our case series, where the operators were more unexperienced than in other studies [14, 16–25]. Moreover, all the patients were treated by different postgraduate students, which can be a determinant factor when making a comparison with other studies and when all the procedures were performed by a single experienced periodontist [14, 17, 19–24].

Another important factor would be the characteristics of the gingival recession at baseline [10, 12, 25], where differences could be found in both depth and width. These factors determine the total avascular area of the treated surface and are directly associated with the difficulty in achieving CRC [8]. In the study by Gil et al. [14], the mean baseline recession was 2.50 mm; in the present study, it was higher at 3.12 mm. In addition, the mean width of recessions should also be considered. In the present sample, where six molars were treated, the mean width of the recessions was 4.35 mm.

In addition, the location of the recession could influence the success of the treatment, as the mandible [37] and the molar and premolar areas [38] would be the locations involving a higher difficulty; in this case series, these areas represented 79% and 58% of the treated recession, respectively. Recently, Gil et al. [14] found an association between the molar location of recessions and lower treatment success rate.

With regard to the type of graft used or the harvesting location, together with the surgical technique, the differences have also been reported. Gil et al. 2018 [14] harvested a CTG either from the palate or the tuberosity or used soft tissue substitutes (acellular dermal matrix allograft or a xenogeneic collagen matrix) with platelet-derived growth factors. This could confound the attribution of the good results to the technique itself or to the type of CTG [39] or biomaterials used [17, 19, 24]. In the present study, CTG was harvested only from the palate, which is still the gold standard for the treatment of gingival recession [8].

Another controversial issue is the thickness of the CTG when treating Miller class III recession [4]. Esteibar et al. [12] showed that a baseline recession width ≤ 3 mm, an interproximal bone loss ≤ 3 mm, and the use of a thick graft (> 2 mm) would increase the likelihood of achieving CRC. However, other authors suggested the use of a thinner graft to aid vascularization [40]. In the present study, a CTG > 2 mm was harvested in all the cases and a gain in the keratinized tissue of 1.11 mm was achieved, which was significantly superior to the 0.5 mm reported by Gil et al. [14]. Thus, the quality of the harvested graft and the coronal traction of the tunnel-papillae-graft complex might have been helpful in achieving CRC in 29% of the treated recessions.

To our knowledge, no study on mucogingival surgery has assessed post-surgical acute pain with a VAS scale at so many follow-up points; however, it has been applied in trials in which impacted wisdom teeth were extracted [41], normally in hospital settings. Due to the difficulty of obtaining information at different time intervals, a specific diary of pain was designed. Post-surgical pain decreased significantly one day after the surgery and did not last for more than a week post the intervention in a majority of patients. In the two cases in which the pain lasted longer, the pain might have been associated with the post-surgical complication (herpes simplex virus) that appeared in one patient or the moderate CSI severity-level score [30] that another patient showed.

Finally, it should be noted that the present study has a few limitations, such as it being a case series with a limited number of patients and recessions. The sample was obtained from a university clinical setting, where the patients’ expectations were not very high. This would also explain the high VAS values observed when assessing the esthetic outcome. In addition, the operators had low surgical experience and the Miller class III recession was difficult to treat; however, reasonable short-term outcomes were achieved.

## Conclusion

Within the limitations of this case series, it can be concluded that the modified VISTA technique offers several advantages in the treatment of Miller class III gingival recession.

More clinical trials with a longer follow-up are needed when Miller class III recessions are treated with different mucogingival surgical techniques that use the same kind of graft and assess the patients’ perception.

## Supplementary Information

**Additional file1.** The pain diary specially designed at our university to register the patients’ perceptions of acute post-surgical pain, for there is no evidence of post-surgical pain in mucogingival surgery during the first 24 h. The UPV/EHU-Pain Diary has two pages. In the first page, all the recordings are registered, and in the second page, there are instructions for the patients, and an explanation of how to fill the pain-diary. The recorded parameters in this *UPV/EHU-Pain Diary* were as follows: highest intensity of perceived pain (measured by a visual analogue scale [VAS]: 0–100 mm), minutes of the duration of this perceived pain, and additional drug intake by the patients apart from the initially prescribed treatment. The UPV/EHU-Pain Diary should be recorded until the pain disappears.

## Data Availability

The datasets generated and/or analyzed during the current study are not publicly available but are available from the corresponding author on reasonable request.

## Abbreviations

CRC: Complete root coverage
RC: Root coverage
RCT: Randomized clinical trial
MRC: Mean root coverage
CAF: Coronally advanced flap
CTG: Connective tissue graft
rhPDGF: Recombinant human platelet-derived growth factor-BB
ADM: Acellular dermal matrix
VISTA: Vestibular incision subperiosteal tunnel access
m-VISTA: Modified VISTA
UPV/EHU: University of the Basque Country
PD: Probing depth
REC: Gingival recession
GRW: Width of the gingival recession
KGW: Width of the keratinized gingiva
CP-IP: Distance from the contact point to the interdental papilla
FMPI: Full mouth plaque index
FMBI: Full mouth bleeding index
CSI: Central Sensitization Inventory
VAS: Visual analogue scale
PSC: Post-surgical complications
FTF: Full thickness flap
e-MCTG: Expanded mesh CTG
CAL: Clinical attachment level
Cig/day: Cigarettes/day
FS: Former smoker
NS: Non-smoker
S: Smoker
Mx: Maxilla
Md: Mandible
SD: Standard deviation
