# Supplementary material for: Description of the modified vestibular incision subperiosteal tunnel access (m-VISTA) technique in the treatment of multiple Miller class III gingival recessions: a case series
Source: BMC Oral Health. 2021 Mar 20;21:142. doi: 10.1186/s12903-021-01511-5 (PMC7981913; doi:10.1186/s12903-021-01511-5)
Supplement: Supplementary file 1 — Additional file1. The pain diary specially designed at our university to register the patients’ perceptions of acute post-surgical pain, for there is no evidence of post-surgical pain in mucogingival surgery during the first 24 h. The UPV/EHU-Pain Diary has two pages. In the first page, all the recordings are registered, and in the second page, there are instructions for the patients, and an explanation of how to fill the pain-diary. The recorded parameters in this UPV/EHU-Pain Diary were as follows: highest intensity of perceived pain (measured by a visual analogue scale [VAS]: 0–100 mm), minutes of the duration of this perceived pain, and additional drug intake by the patients apart from the initially prescribed treatment. The UPV/EHU-Pain Diary should be recorded until the pain disappears. [file 12903_2021_1511_MOESM1_ESM.pdf]

UPV/EHU-Pain Diary

3 days, every 8 hours (1st day: 2 and 4 hours) with the antibiotic intake. Then daily, late in the day, until total remission of pain

Pre-surgery		2 h	4 h	8 h	16 h
**Head-neck pain**	**At this moment**				
In the last month?	Data: _____/_____/______	Data: _______/______/_____<br>Time:________________	Data: ___/______/_____<br>Time:________________	Data: ___/______/_____<br>Time:________________	Data: ___/______/_____<br>Time:________________
1.Yes: Intensity (0-100 scale)<br>__________________________<br>2.No	1.Yes: Intensity (0-100 scale)<br>__________________________<br>2.No	1.Intensity (0-100):________<br>2.Duration (min/h):________<br>3.Treatment (yes/no; which):<br>__________________________	1.Intensity:______________<br>2.Duration:______________<br>3.Treatment:____________<br>__________________________	1.Intensity:______________<br>2.Duration:______________<br>3.Treatment:____________<br>__________________________	1.Intensity:______________<br>2.Duration:______________<br>3.Treatment:____________<br>__________________________
**24 h (1st day)**	**32 h**	**40 h**	**48 h (2nd day)**	**56 h**	**64 h**
Data: ___/______/_____<br>Time:________________	Data: ___/______/_____<br>Time:________________	Data: ___/______/_____<br>Time:________________	Data: ___/______/_____<br>Time:________________	Data: ___/______/_____<br>Time:________________	Data: ___/______/_____<br>Time:________________
1.Intensity:______________<br>2.Duration:______________<br>3.Treatment:____________<br>__________________________	1.Intensity:______________<br>2.Duration:______________<br>3.Treatment:____________<br>__________________________	1.Intensity:______________<br>2.Duration:______________<br>3.Treatment:____________<br>__________________________	1.Intensity:______________<br>2.Duration:______________<br>3.Treatment:____________<br>__________________________	1.Intensity:______________<br>2.Duration:______________<br>3.Treatment:____________<br>__________________________	1.Intensity:______________<br>2.Duration:______________<br>3.Treatment:____________<br>__________________________
**72 hs (3rd day)**	**4th day**	**5th day**	**6th day**	**7th day**	**8th day**
Data: ___/______/_____<br>Time:________________	Data: ___/______/_____<br>Time:________________	Data: ___/______/_____<br>Time:________________	Data: ___/______/_____<br>Time:________________	Data: ___/______/_____<br>Time:________________	Data: ___/______/_____<br>Time:________________
1.Intensity:______________<br>2.Duration:______________<br>3.Treatment:____________<br>__________________________	1.Intensity:______________<br>2.Duration:______________<br>3.Treatment:____________<br>__________________________	1.Intensity:______________<br>2.Duration:______________<br>3.Treatment:____________<br>__________________________	1.Intensity:______________<br>2.Duration:______________<br>3.Treatment:____________<br>__________________________	1.Intensity:______________<br>2.Duration:______________<br>3.Treatment:____________<br>__________________________	1.Intensity:______________<br>2.Duration:______________<br>3.Treatment:____________<br>__________________________
**9th day**	**10th day**	**11th day**	**12th day**	**13th day**	**14th day**
Data: ___/______/_____<br>Time:________________	Data: ___/______/_____<br>Time:________________	Data: ___/______/_____<br>Time:________________	Data: ___/______/_____<br>Time:________________	Data: ___/______/_____<br>Time:________________	Data: ___/______/_____<br>Time:________________
1.Intensity:______________<br>2.Duration:______________<br>3.Treatment:____________<br>__________________________	1.Intensity:______________<br>2.Duration:______________<br>3.Treatment:____________<br>__________________________	1.Intensity:______________<br>2.Duration:______________<br>3.Treatment:____________<br>__________________________	1.Intensity:______________<br>2.Duration:______________<br>3.Treatment:____________<br>__________________________	1.Intensity:______________<br>2.Duration:______________<br>3.Treatment:____________<br>__________________________	1.Intensity:______________<br>2.Duration:______________<br>3.Treatment:____________<br>__________________________

1. **Intensity (worst pain):** On this scale from 0 "no pain" to 100 "the worst pain you can imagine" (VAS)

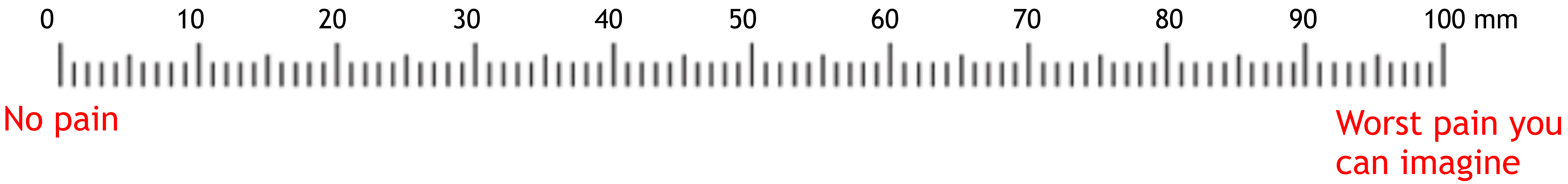

In the scale please choose, where is the maximum perceived pain in this period of time?

2. **Duration:**

What has been the duration, in minutes or hours, of the pain experienced in this stretch of time?

3. **Treatment:**

Have you needed any additional analgesic treatment during this period of time?

No or Yes: Which one?
